# Metal backed fixed-bearing unicondylar knee arthroplasties using minimal invasive surgery: a promising outcome analysis of 132 cases

**DOI:** 10.1186/s12891-015-0651-x

**Published:** 2015-07-31

**Authors:** Joel Baur, Lukas Zwicky, Michael Tobias Hirschmann, Thomas Ilchmann, Martin Clauss

**Affiliations:** Kantonsspital Baselland, Clinics for Orthopaedics and Trauma Surgery, Rheinstrasse 26, CH-4410 Liestal, Switzerland; Leonardo - Klinik Birshof, CH-4142 Muenchenstein, Switzerland

## Abstract

**Background:**

Unicondylar knee arthroplasty (UKA) is a well-established treatment for isolated osteoarthritis (OA) of the medial knee compartment. Aim of this retrospective study was to evaluate the early clinical and radiological outcomes of a consecutive series of patients treated with medial metal backed fixed-bearing UKA. Furthermore, the influence of the component orientation on the outcome was analyzed.

**Methods:**

From 09/2006 to 11/2010 106 patients (132 knees; 69 ± 9 years) were treated using a metal backed fixed-bearing UKA with a MIS approach. All patients underwent a standardized clinical and radiological follow-up at 6 weeks, 1, 2 and 5 years. Mean follow-up was 3.4 ± 1.0 years. Two patients (three UKAs) deceased and two patients (two UKAs) were lost to follow-up. Three different survival analyses were performed using three different endpoints defining failure: (a) revision with exchange of any UKA component (b) aseptic loosening and (c), a worst case scenario, where it was assumed that all progressive radiolucencies would lead to aseptic loosening and thus these were additionally counted. Clinical outcome was assessed using the American knee society score (AKS) and the Oxford knee score (OKS). Radiographic analysis was done according to the American Knee Society Evaluation and Scoring System adapted for UKA and correlated with the AKS and OKS.

**Results:**

Five UKAs (3.8 %) were revised to total knee arthroplasties (TKAs) after a median of 25 (10–33) months. Five year survival was 95.2, 97.5 and 87.7 % for the aforementioned endpoints. At final follow-up the median AKS knee score was 99 (50–100) points and the median AKS function score was 100 (60–100) points. The median OKS was 43 (8–48) points. Clinical outcome was independent of the component orientation.

**Conclusion:**

Fixed-bearing UKA showed excellent clinical and radiological results at up to 5 years follow-up. Outcome was independent of component orientation.

## Background

Unicondylar knee arthroplasty (UKA) is a well-established surgical option for isolated medial unicompartimental arthritis which has shown excellent early results [9]. Despite advanced instrumentation systems UKA is still a challenging procedure. Although minimal invasive surgical (MIS) techniques for UKA have gained popularity in the last decade [22], they may result in inferior component alignment, a prolonged learning curve and increased early failure rates [22].

There are two different designs (fixed-bearing vs. mobile-bearing prosthesis) which have shown comparable clinical and radiological outcome, quality of life and revision rates [6, 28]. However, mobile-bearing UKAs have shown more early failures while metal-backed fixed-bearing UKAs have shown higher long-term failure rates due to increased polyethylene wear [6]. All-poly designs have shown inferior five-year survival rates when compared to the metal back designs [35]. During the last years mobile-bearing UKA have become increasingly popular because they are considered to recreate native knee kinematics more closely [2, 12]. Furthermore, they have a bigger acceptable range of component alignment compared to fixed-bearing UKA [11]. However, their major disadvantage is a substantially longer learning curve [6, 28].

The aim of this retrospective study was to evaluate the clinical, subjective and radiological outcomes of the first 132 consecutively implanted medial UKAs using a metal backed fixed-bearing with a MIS approach at a university affiliated teaching hospital. Special interest was paid to the question whether component orientation correlates with early functional, subjective and radiological outcomes or even higher revision rates.

## Methods

### Study design

From September 2006 to November 2010 a total of 132 medial UKAs were consecutively implanted in 106 patients at our institution. The study group consisted of 53 men (65 knees) and 53 women (67 knees). Half of the surgeries (66 UKAs) were performed on the left and the other half on the right knee, 26 patients received a UKA on both sides, 20 of them in a single surgery. The average age of the patients at surgery was 69 ± 9 years. Patients with other implants such as total knee arthroplasties (TKA, *n* = 658) or lateral UKA (*n* = 4) operated in the study period were not included in the study. The study was conducted in accordance with good clinical practice and ethical approval was obtained Ethikkomission Nordwest und Zentralschweiz (EKNZ 2015-229). All patients gave informed consent for the study. All patients were followed prospectively at six weeks, one, two and five years after surgery according to the in-house register documentation [3]. Data analysis was performed retrospectively. The mean follow-up was 3.4 ± 1.0 years.

### Implant

The Zimmer Unicompartmental High Flex Knee System™ (ZUK; Zimmer, Winterthur, Switzerland) is a fixed-bearing metal back UKA developed from the M/G Unicompartmental Knee System™ (Zimmer, Winterthur, Switzerland) [5] and can be implanted with a MIS approach (spacer-block technique). However, clinical and radiological data for this new implant system is scarce [27].

### Indication, surgical technique and early postoperative care

Medial compartment arthritis was diagnosed on conventional (weight-bearing short anterior-posterior (ap), true lateral, sunrise view and full-length hip-knee-ankle (HKA)) radiographs. The function of the anterior cruciate ligament (ACL) was confirmed prior to surgery by Lachman testing and in doubt an MRI was performed to confirm ACL integrity. In case of an insufficient ACL a TKA was implanted. A tourniquet control was used in all cases. Surgery was performed or supervised by a team of nine different orthopaedic consultants. A MIS medial parapatellar approach, incising only the medial capsule from the medial patella pol down to the tibial tuberosity [1], was used combined with the spacer-block technique. All components were cemented using Palacos R + G bone cement (Haereus Medical, Weinheim, Germany). Postoperatively a continuous passive mobilization machine was used and full weight bearing exercises were initiated from the first postoperative day under guidance of a physical therapist.

### Survival analysis

Three different survival analyses were performed using Kaplan-Meier analysis with three different endpoints defining failure: (a) revision with exchange of any component for any reason, (b) revision due to aseptic loosening and (c), a worst case scenario, where all cases with progressive radiolucencies were additionally counted to the cases with aseptic loosening because they were assumed to lead to aseptic loosening. Revision was defined as an exchange, addition or removal of any component for any reason, reoperation was defined as any intervention even without exchanging any of the components [3]. Any complications related to the implant were prospectively recorded. Clinical records were screened for additional information.

### Clinical outcome

Clinical outcome was prospectively assessed using the American Knee Score (AKS) at 6 weeks, 1, 2 and 5 years [8].

The AKS is comprised of two parts: The knee score addressing pain, stability and range of movement (ROM) and the function score which examines function, with particular reference to stair climbing, walking distance or whether walking aids are needed. For each section, the maximum score is 100 points [7].

Furthermore an Oxford Knee Score (OKS) was assessed during the 5-year follow-up control [7]. For patients with a follow-up less than 5 years the OKS was completed during a telephone interview. For the OKS each question is weighted between zero and four, with four being the best outcome, leading to an overall possible score between 0 and 48 points [25].

### Radiological outcome

Radiological follow-up was scheduled according to a standardized prospective protocol at 6 weeks, 1, 2 and 5 years of follow-up including weight-bearing short anterior-posterior (ap), true lateral and patella sunrise view radiographs [3]. Additional full-length hip-knee-ankle (HKA) radiographs were performed preoperatively as well as one and five years postoperatively. The mean radiological follow-up was 2.5 ± 1.2 years.

**Fig. 1:**
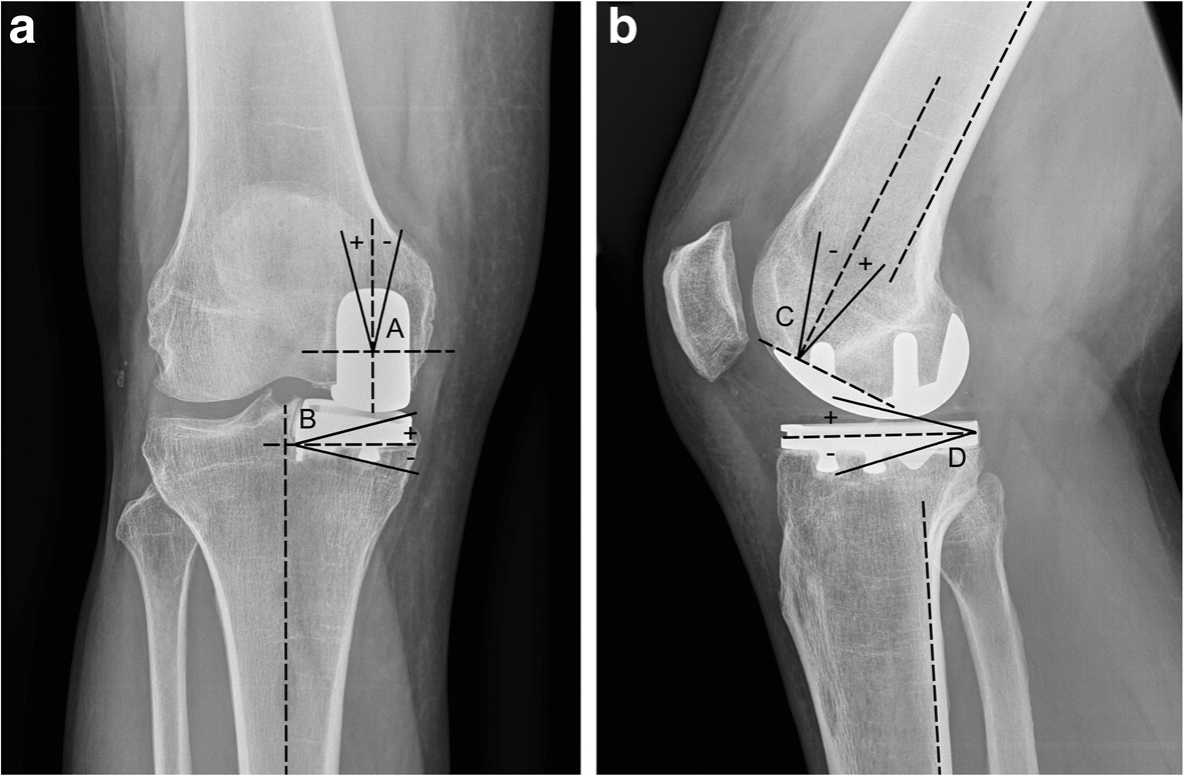
Radiograph of the lower extremity of a 68 year old male patient with a ZUK implanted, illustrating the measurement of the femoral condyle angle (**a**), the tibial plateau angle (**b**), the femoral flexion (**c**) and the tibial tilt (**d**). Positive values are defined as valgus, extension or positive tilt

Radiographic evaluation was performed as described by Sarmah et al. [31] (Fig. 1). In detail, we measured the alignment of the femoral component to the femoral axis in the ap (A) and lateral radiographs (C) as well as the alignment of the tibial component to the tibial axis in the ap (B) and lateral radiographs (D). Changes in the alignment of the component between the first and last postoperative radiographs were calculated. Additionally, the mechanical femoral axis of the leg was measured on the HKA radiograph. All measurements were performed by one independent observer (JB) within a month using digital measurement software (ims, Imagic Bildverarbeitung AG, Glattbrugg, Switzerland). To control the intraobserver reliability one independent observer (JB) remeasured ten randomized blinded radiographic series.

**Fig. 2:**
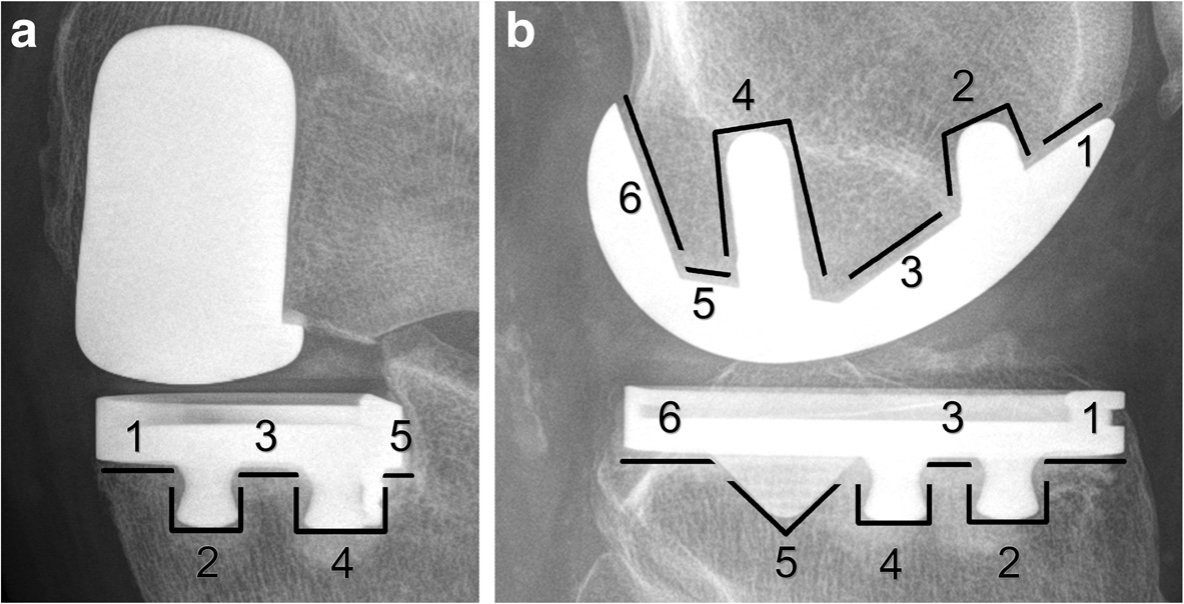
Anterior-posterior (**a**) and lateral (**b**) radiographs of a 92 year old male patient with the ZUK in situ illustrating the radiolucent zones which were used in the present study

Radiolucencies were divided into non-progressive and progressive radiolucencies. The non-progressive radiolucencies were defined as (1) less than two millimeters thick, (2) well defined and (3) with a parallel radiodense line. The progressive radiolucencies were defined (1) as thicker than two millimeters and (2) not well defined [31]. The radiolucent zones were classified as defined by Kalra et al. [17] and adapted to the UKA implant (Fig. 2). All radiographs were examined for progressive and non-progressive radiolucencies by two reviewers (JB and MC) and were defined as a consensus if both found radiolucencies. Finally all radiographs were checked for progressive osteoarthritis (OA) in the lateral or patellofemoral knee compartment as defined by Kellgren and Lawrence [19].

### Statistics

Statistical analysis was performed using IBM SPSS Statistics 21 (IBM Corporation, Somers, New York). A Shapiro-Wilk test was performed to verify normal distribution of the data. A dependent-sample *t*-test was used to compare paired metric parameters, a Wilcoxon test for not normally distributed data.

To compare outcome parameters in two groups either the independent-sample *t*-test or the Mann-Whitney-Test was performed. Correlation between ordinal-scaled parameters was determined using Spearman’s correlation. A Chi-square test was used to compare nominal data between two groups. The level of significance was set at *p* < 0.05.

For the intra-observer reliability of the performed radiological values, the two-way random intraclass correlation coefficient (ICC 2,1) with single measurement and absolute agreement were calculated for each parameter and presented with 95 % confidence interval. The interpretation of the ICC values was graded using the classification scheme of Munro, as low (0.26–0.49), moderate (0.50–0.69), high (0.70–0.89) and very high (0.90–1.00) [23].

The radiological and demographic data were expressed as a mean value ± standard deviation if the data was normally distributed, if not we used median and range.

## Results

Two patients (three UKAs) deceased unrelated to the index surgery and one patient (two UKAs) was unable to attend the follow-up due to medical illness unrelated to UKA. None of these patients underwent any revisions or reoperations. Two UKAs (1.5 %, two patients) of the 132 were lost to follow-up.

Five UKAs (3.8 %) were revised to a TKA after a median of 25 (10–33) months following index surgery (Table 1). One UKA had debridement, exchange of the inlay and retention of the implant due to infection (*S. aureus*) eight months postoperatively [37]. Another six UKAs were revised without exchange of the components (median 4 months; range 6 days to 33 months, Table 2).

**Table 1:** Revision to TKA

Indication for revision to TKA	n (%)	Time from surgery (months)
Aseptic loosening of tibial component	3 (2.3 %)	25, 30, 33
ACL rupture (instability)	1 (0.8 %)	10
Infection	1 (0.8 %)	19

**Table 2:** Reoperations

Indication for reoperation	n (%)	Treatment	Time from surgery (months)
Persistent knee problems	2 (1.5 %)	Knee arthroscopy	2, 20
Arthrofibrosis	2 (1.5 %)	Knee arthroscopy	33
		Mobilisation in anaesthesia	1
Infection	1 (0.8 %)	Debridement, inlay exchange	8
Haematoma	1 (0.8 %)	Debridement	0.2
Wound healing disorder	1 (0.8 %)	Debridement	6

### Survival analysis

**Fig. 3:**
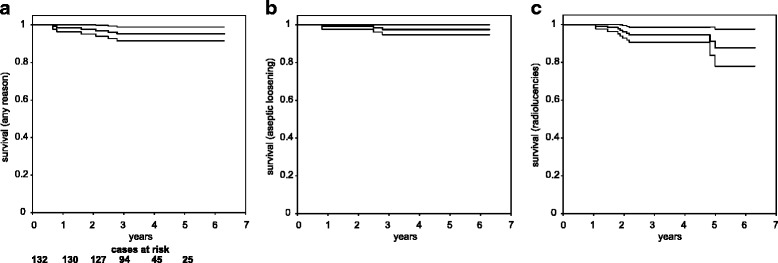
Survival analysis with the endpoints: (**a**) revision with exchange of any component for any reason, (**b**) revision due to aseptic loosening (**c**) a worst case scenario counting all cases with aseptic loosening and the UKA with progressive radiolucencies as failure

The survival rate after five years with the endpoint exchange of any UKA component was 95.2 % (95 % Confidence Interval (CI) 91.5–98.9 %; Fig. 3a), the survival rate for revision due to aseptic loosening was 97.5 % (95 % CI 94.8–100 %; Fig. 3b) and the survival rate for revision due to aseptic loosening calculated assuming the worst case scenario where all the UKAs with progressive radiolucencies would need a revision was 87.7 % (95 % CI 77.9–97.5 %; Fig. 3c).

### Clinical results

The change of the AKS subscores over the years is shown in Table 3. At final follow-up, the median AKS knee score was 99 (50–100) points and the median AKS function score was 100 (60–100) points. The median ROM was 130° (95°–150°) flexion and 0° (−10°–5°) extension.

**Table 3:** Development of the AKS scores during follow-up

Follow-up	Knee Society knee score	Knee Society function score
6 weeks	92 (38–100)	90 (60–100)
1 year	95 (60–100)	100 (40–100)
2 years	95 (44–100)	100 (40–100)
5 years	99 (50–100)	100 (60–100)

Data presented as median (range)

The median OKS at final follow-up was 43 (8–48) points.

### Radiological results

Lateral and ap radiographs were available for all UKAs, but 14 UKAs had a missing preoperative and 13 a missing postoperative HKA radiograph.

We found no progressive osteoarthritis in any of the patients as defined by Kellgren and Lawrence [19].

The angles which were measured are listed in Table 4. UKA component alignment in patients with aseptic loosening did not change postoperatively and was not different to patients without loosening.

**Table 4:** Pre- and postoperative alignment of the lower extremity and component alignment of the ZUK in the first and last postoperative anterior-posterior (ap) and lateral radiographs

	ICC	Preoperative	First postoperative	Last postoperative	*P* value	n
Mechanical axis	0.98 (0.94–0.97)	−1.4 (1.9, −5.8–4.9), *n* = 118	−0.3 (1.8, −6.0–3.9), *n* = 119		<0.001	110
A	0.45 (−0.11–0.82)		−4.0 (4.9, −18.6 –9.2), *n* = 131	−4.8 (4.9, −19.5–9.3), *n* = 131	0.002	131
B	0.65 (0.13–0.90)		-2.2, (3.3, −9.6–3.9), *n* = 122	−1.8 (3.2, −9.5–5.8), *n* = 122	0.115	114
C	0.96 (0.84–0.99)		1.0 (5.3, −12.3–15.5), *n* = 132	0.9 (5.2, −12.3–14.7), *n* = 132	0.667	132
D	0.95 (0.83–0.99)		3.3 (2.9, −2.9–9.6), *n* = 131	3.5 (2.8, −3.8–9.9), *n* = 132	0.374	131

*ICC* Intraclass correlation coefficient, *A* femoral component to the femoral axis ap, *B* tibial component to the tibial axis ap, *C* femoral flexion lateral, *D* tibial tilt lateral

Data are presented as mean (standard deviation, range) or 95 % confidence intervall (ICC). Positive values are defined as valgus, extension or positive tilt

Radiolucencies were found in 44 UKAs (33 %), seven (5 %) of these 44 UKAs had progressive radiolucent lines (Table 5, Fig. 2). Comparison of UKA component position in patients with progressive radiolucent lines in serial imaging and patients without radiolucent lines, did not reveal any significant difference.

**Table 5:** Distribution of radiolucencies according to the determined zones (Fig. 2)

		Zone 1	Zone 2	Zone 3	Zone 4	Zone 5	Zone 6
Femoral (lateral view)	non-progressive	1 (1.9 %)	0 (0.0 %)	8 (15.1 %)	0 (0.0 %)	0 (0.0 %)	9 (17.0 %)
	progressive	0 (0.0 %)	0 (0.0 %)	3 (15.8 %)	0 (0.0 %)	0 (0.0 %)	0 (0.0 %)
Tibial (lateral view)	non-progressive	12 (22.6 %)	0 (0.0 %)	1 (1.9 %)	0 (0.0 %)	1 (1.9 %)	11 (20.8 %)
	progressive	2 (10.5 %)	1 (5.3 %)	1 (5.3 %)	1 (5.3 %)	2 (10.5 %)	3 (15.8 %)
Tibial (anterior-posterior view)	non-progressive	8 (15.1 %)	1 (1.9 %)	0 (0.0 %)	0 (0.0 %)	1 (1.9 %)	
	progressive	2 (10.5 %)	1 (5.3 %)	1 (5.3 %)	1 (5.3 %)	1 (5.3 %)	

No difference in component positioning was found in patients with an AKS of less than 90 points (*n* = 24) compared to those greater than 90 points. Comparing the component position in UKAs with an OKS less than 40 points (*n* = 43) to those greater than or equal to 40 points, showed that only the angle B (1°, *p* = 0.036), was significantly different between the two groups. Furthermore the radiological alignment did not correlate with the clinical instability assessed using the AKS.

There were no UKAs with tibial component malalignment (>10° in any direction). The UKAs with femoral component malalignment > 10° (*n* = 12) did not have a significant different ROM (*p* = 0.582) nor did they have a significant different rate of the progression of radiolucencies (*p* = 0.326), when compared to the UKAs with neutrally implanted femoral components.

UKAs with a slight overcorrection of the mechanical axis from varus to valgus alignment (*n* = 26) showed no difference in the OKS (*p* = 0.597), the AKS (*p* = 0.853) and in the rate of progressive radiolucent zones (*p* = 0.844).

## Discussion

The study showed an excellent 5-year survival rate of 97.2 % for aseptic loosening. This is comparable to the literature of other successful implant designs [20] and the data shown by the 2011 Australian and Swedish National Registers for the ZUK [9, 34]. In addition, excellent clinical and functional outcomes were found. This confirms the results from the only other study available for this implant system [27] and is comparable to the literature of other established implant designs [5, 10, 20, 21, 26, 29]. With only two UKAs (1.5 %) lost to follow-up, data should be unbiased [24].

Radiological alignment in our series was within the normal range of high volume UKA centers [30] using a MIS approach. Hospitals with low numbers of UKAs have a higher failure rate than the ones with higher numbers [4, 30]. The operations in our study were carried out or supervised by nine different consultants, the results might even be better if fewer surgeons would have performed the same amount of UKAs [4, 30]. For UKAs, component alignment is believed to be mandatory for good long-term survival and clinical outcome, for both fixed [18] and mobile-bearing UKAs [16, 18, 33]. Up until the time point of the 5-year follow-up, we were unable to find any correlation between component alignment and clinical outcome, contradictory to the literature. A further aspect is that due to the excellent 5-year implant survival (97.2 %) for aseptic loosening, the study might be underpowered to find relevant differences, especially with the low variation of component orientation. Another explanation might be that the AKS and OKS may not be accurate enough to detect small clinical differences.

Aim of UKA implantation is to correct only the wear deformity restoring the original mechanical axis [27, 36], thus overstuffing of the lateral or patellofemoral compartment can be avoided. Analyzing the alteration of the mechanical axis we had 26 slightly overcorrected UKAs. However, overcorrection in the short-term had no influence on progression of OA in the other compartments, but this remains of major concern for further follow-up.

Overall 44 UKAs in our series showed radiolucent lines. The vast majority of the radiolucencies (93 %) were non-progressive and mostly situated at the edge of the components (posterior femoral condyle, posterior and anterior on the tibial side). They were most likely due to an insufficient cementing technique and thus their frequency could have been reduced with a more advanced cementing technique and with the use of a jet lavage system [14, 15, 32]. Of concern are the seven UKAs with progressive radiolucencies at the time of final follow-up. Whether these will end up in aseptic loosening or not in the future is unclear and must be monitored.

### Limitations

The major limitation of this study is that our mean follow-up of 3.4 years is still rather short but, to our knowledge, clinical and radiological mid- and long-term data for the investigated implant system are not yet available. Furthermore, data analysis was done retrospectively lacking a control group with a non MIS UKA.

We used plain radiographs and the “Knee Society radiological evaluation and scoring system” [8] as an accepted tool for radiological analysis [3, 13, 31]. Plain radiographs are the most commonly used modality to measure component alignment, although gold standard is 3-dimensional computed tomography (3D-CT) [13, 31]. However, the use of 3D-CT is not practicable in bigger study groups. The achieved intra-observer ICC showed a very good reproducibility of the measurements on lateral radiographs (C, D) and for the mechanical axis measured on the HKA radiographs, but only a low to moderate reliability of the measurements on the ap radiographs.

## Conclusion

Survival, functional and radiological results at the time point of up to 5 years of follow-up were excellent. Component alignment was not influenced by the MIS technique used and with the limitation of the given measurement accuracy obtained from plain radiographs, component alignment did not influence functional outcome. However, mid- and long-term data are necessary to analyze the longevity of this new implant system.

## Abbreviations

UKA: Unicondylar knee arthorplasty
MIS: Minimal invasive surgical
TKA: Total knee arthoplasty
ZUK: Zimmer Unicompartmental High Flex Knee System
ACL: Anterior cruciate ligament
AKS: American Knee Score
ROM: Range of movement
OKS: Oxford Knee Score
OA: Osteoarthritis
ap: Anterior-posterior
HKA: Hip-knee-ankle
ICC: Intraclass correlation coefficient
CI: Confidence interval
3D-CT: 3-dimensional computed tomography
